# Supplementary material for: Understanding In Vitro Pathways to Drug Discovery for TDP-43 Proteinopathies
Source: Int J Mol Sci. 2022 Nov 25;23(23):14769. doi: 10.3390/ijms232314769 (PMC9738080; doi:10.3390/ijms232314769)
Supplement: Supplementary file 1 [file ijms-23-14769-s001.zip › ijms-1985492-supplementary.pdf]

Supplementary Material

Understanding in vitro pathways to drug discovery for TDP-43 proteinopathies

Hei W.A. Cheng[‡, 1], Timothy B. Callis[‡, 2], Andrew P. Montgomery[2], Jonathan J. Danon[2], William T. Jorgensen[2], Yazi D. Ke[3], Lars M. Ittner[3], Eryn L. Werry[2,4], Michael Kassiou[2,*]

1. School of Medical Sciences, Faculty of Medicine and Health, The University of Sydney, NSW, 2006, Australia
2. School of Chemistry, Faculty of Science and the University of Sydney, NSW, 2006, Australia
3. Department of Biomedical Sciences, Faculty of Medicine and Health Sciences, 2 Technology Place, Macquarie University, NSW, 2109, Australia
4. Central Clinical School, Faculty of Medicine and Health, The University of Sydney, NSW, 2006, Australia

* Correspondence: michael.kassiou@sydney.edu.au

Contents

General Experimental Details 2

Synthesis and Characterisation of 1 3

Synthesis and Characterisation of 2 5

References: 7

General Experimental Details

Unless otherwise stated, all solvents and reagents were purchased and used from commercial sources. Anhydrous solvents were obtained from an Innovative Technology PureSolv7 purification system.

Nuclear magnetic resonance spectra were recorded at 300 K using either a Bruker AVANCE DRX300 (300 MHz), AVANCE DRX400 (400 MHz) or AVANCE DRX500 (500 MHz) spectrometer. ^{1}H chemical shifts are expressed as parts per million (ppm) with residual chloroform (δ 7.26), methanol (δ 3.31) or dimethyl sulfoxide (δ 2.50). ^{13}C chemical shifts are expressed as parts per million (ppm) with residual chloroform (δ 77.16), methanol (δ 49.00) or dimethyl sulfoxide (δ 39.52).

Low-resolution mass spectra (LRMS) were recorded using electrospray ionisation (ESI) recorded on a Bruker AmaZon SL ion trap spectrometer. High-resolution mass spectrometry (HRMS) was performed on a Bruker Apex Qe 7T Fourier Transform Ion Cyclotron Resonance mass spectrometer equipped with an Apollo II ESI/MALDI dual source.

High performance liquid chromatography (HPLC) analysis of organic purity was conducted on a Waters Alliance 2695 instrument using a SunFire™ C18 column (5 µm, 2.1 x 150 mm) and detected using a Waters 2996 photodiode array (PDA) detector set at either 230 or 254 nm. Separation was achieved using water + 0.1% trifluoroacetic acid (solvent A) and acetonitrile + 0.1% trifluoroacetic acid (solvent B) at a flow rate of 0.2 mL/min and a gradient of 0% B to 100% B over 30 minutes. HPLC data is reported as percentage purity and retention time (R_T) in minutes.

Synthesis and Characterisation of 1

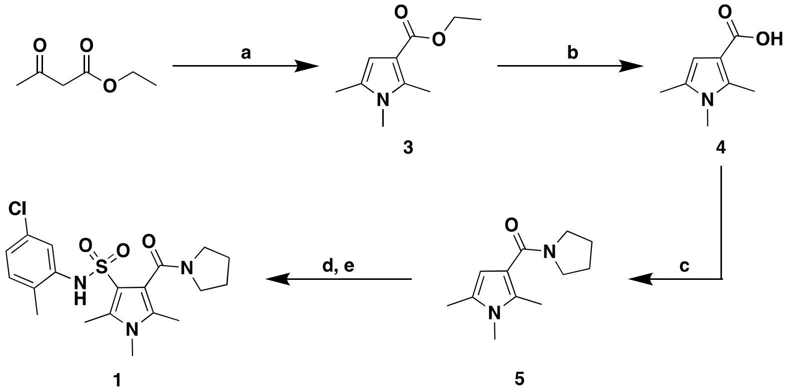

Scheme S1. Synthesis of reported TDP-43 aggregation inhibitor **1**. Reagents and Conditions: (a) i) Chloroacetone, NaI, Acetone, rt, 30 min ii) Methylamine, CAN, $AgNO_3$, MeOH, rt, 6 h, 56% (b) $NaOH_{(aq)}$, EtOH, rt, 12 h, 74% (c) Pyrrolidine, PyBOP®, $^{i}Pr_2EtN$, DMF, rt, 1 h, 46% (d) HSO_3Cl, MeCN, 1 h, 0 °C (e) 5-Chloro-2-methylaniline, Et_3N, MeCN, 0 °C – rt, 1 h, 27% (over 2 steps).

Ethyl 1,2,5-trimethyl-1*H*-pyrrole-3-carboxylate (3)

A solution of NaI (3. 47 g, 23.2 mmol) in acetone (40 mL) was treated with 1-chloropropan-2-one (1.72 mL, 21.4 mmol) and stirred at rt for 15 min at which point a white precipitate had formed. The reaction mixture was filtered over Celite® to remove the precipitate and the filtrate was then concentrated under reduced pressure. The formed 1-iodopropan-2-one was used without any further purification. A separate solution of ceric ammonium nitrate (400 mg, 730 μmol), ethyl 3-oxobutanoate (1.88 mL, 14.7 mmol) and methylamine (2 M Solution in THF, 9.5 mL, 19.0 mmol) in MeOH (40 mL) was stirred at rt for 1 h. The reaction mixture was then treated with $AgNO_3$ (2.50 g, 14.7 mmol) and stirred until all $AgNO_3$ had dissolved. The crude 1-iodopropan-2-one in MeOH (10 mL) was added to the reaction mixture and stirred at rt for 1.5 h. The reaction was filtered over Celite®, and the filtrate was concentrated under reduced pressure. The crude product was then partitioned between H_2O (50 mL) and CH_2Cl_2 (50 mL). The aqueous phase was washed with CH_2Cl_2 (2 x 50 mL). The combined organic layers were then washed with brine (1 x 50 mL), dried over $MgSO_4$ and concentrated under reduced pressure. The crude product was purified through flash chromatography (1:9 EtOAc/Hex) to yield the title compound as a yellow oil that solidified on standing to give a yellow solid (1.49 g, 56%). **^{1}H NMR** (500 MHz, $CDCl_3$) δ 6.25 (q, *J* = 1.0 Hz, 1H), 4.24 (q, *J* = 7.1 Hz, 2H), 3.39 (s, 3H), 2.50 (s, 3H), 2.18 (d, *J* = 0.9 Hz, 3H), 1.32 (t, *J* = 7.1 Hz, 3H) ppm; **^{13}C NMR** (126 MHz, $CDCl_3$) δ 165.87, 135.55, 127.89, 110.66, 107.20, 59.21, 30.35, 14.70, 12.42, 11.58 ppm**.** Matched previously reported characterisation data [1].

1,2,5-Trimethyl-1*H*-pyrrole-3-carboxylic acid (4)

A solution of **3** (405 mg, 2.24 mmol) in EtOH (10 mL) was treated with NaOH (2 M aq. sol, 5.6 mL, 11.2 mmol) and heated at reflux overnight, at which point TLC indicated conversion of the starting material. The reaction mixture was then diluted with water and acidified to pH 2. The aqueous phase was then extracted with CH_2Cl_2 (3 x 50 mL). The combined organic phases were then washed with brine (1 x 50 mL), dried over $MgSO_4$ and concentrated under reduced pressure to yield the title compound as a white solid (253 mg, 74%). **R_f** 0.58 (1:1 EtOAc/Hex); **^{1}H NMR** (500 MHz, DMSO-d_6) δ 11.43 (s, 1H), 6.04 (q, *J* = 1.0 Hz, 1H), 3.36 (s, 3H), 2.41 (s, 3H), 2.12 (d, *J* = 1.0 Hz, 3H) ppm; **^{13}C NMR** (126 MHz, DMSO-d_6) δ 166.25, 134.58, 127.31, 110.06, 106.96, 30.03, 11.93, 11.07 ppm; **LRMS (+ESI)** m/z: 154 ($[M+H]^+$, 22), 176 ($[M+Na]^+$, 100).

Pyrrolidin-1-yl(1,2,5-trimethyl-1*H*-pyrrol-3-yl)methanone (5)

A solution of **4** (206 mg, 1.35 mmol), PyBOP (757 mg, 1.45 mmol) and pyrrolidine (0.12 mL, 1.44 mmol) in DMF (5 mL) was treated with DIPEA (0.50 mL, 2.88 mmol). The reaction mixture was stirred at rt for 2 h at which point TLC indicated conversion of the starting material. The reaction mixture was then diluted in H_2O (50 mL) and extracted with EtOAc (3 x 50 mL). The combined organic phases were then washed with H_2O (1 x 50 mL), brine (1 x 50 mL), dried over $MgSO_4$ and concentrated under reduced pressure. The crude product was purified through flash chromatography (EtOAc) to yield the title compound as an off-white solid (128 mg, 46%). **^{1}H NMR** (500 MHz, $CDCl_3$) δ 5.97 (q, *J* = 0.9 Hz, 1H), 3.61 – 3.54 (m, 4H), 3.37 (s, 3H), 2.40 (s, 3H), 2.18 (d, *J* = 0.8 Hz, 3H), 1.92 – 1.84 (m, 4H) ppm; **^{13}C NMR** (126 MHz, $CDCl_3$) δ 167.16, 131.89, 126.71, 114.95, 105.64, 49.20, 46.11, 30.17, 26.66, 24.52, 12.48, 11.72 ppm; **LRMS (+ESI)** m/z: 207 ($[M+H]^+$, 34), 229 ($[M+Na]^+$, 94).

***N*-(5-Chloro-2-methylphenyl)-1,2,5-trimethyl-4-(pyrrolidine-1-carbonyl)-1*H*-pyrrole-3-sulfonamide (1)**

A solution of **5** (117 mg, 568 μmol) in MeCN (5 mL) was cooled to 0 °C and put under an atmosphere of N_2. The reaction mixture was then treated dropwise with chlorosulfuric acid (0.38 mL, 5.68 mmol). The reaction mixture was stirred at 0 °C for 1 h at which point TLC indicated conversion of starting material. The reaction mixture was then treated dropwise with Et_3N (0.87 mL, 6.24 mmol) at 0 °C and stirred for a further 30 min. A solution of 5-chloro-2-methylaniline (96 mg, 680 μmol) in MeCN (3 mL) was then added to the reaction mixture and warmed to rt. The reaction mixture was stirred for 1 h and then concentrated under a stream of N_2. The crude reaction mixture was purified by flash chromatography (1:1 EtOAc/Hex) to yield the title compound as a white solid (63 mg, 27%). **R_f** 0.45 (EtOAc); **^{1}H NMR** (300 MHz, $CDCl_3$) δ 7.49 (s, 1H), 7.25 (d, *J* = 2.1 Hz, 1H), 7.03 (d, *J* = 8.2 Hz, 1H), 6.97 (dd, *J* = 8.2, 2.1 Hz, 1H), 3.87 – 3.71 (m, 1H), 3.57 – 3.39 (m, 2H), 3.35 (s, 3H), 3.30 – 3.13 (m, 1H), 2.31 (s, 3H), 2.19 (s, 3H), 2.17 (s, 3H), 2.08 – 1.81 (m, 4H) ppm; **^{13}C NMR** (126 MHz, $CDCl_3$) δ 166.78, 137.66, 133.78, 131.63, 131.25, 131.22, 126.71, 125.09, 123.09, 116.32, 114.63, 48.74, 45.96, 30.73, 25.85, 24.84, 17.75, 11.47, 10.34 ppm; **LRMS (+ESI)** m/z: 432 ($[M+Na]^+$, 100), 841 ($[2M+Na]^+$, 10); **HRMS (+ESI)** Calc. for $C_{19}H_{24}ClN_3O_3S$ $[M+Na]^+$: 432.1125/434.1095, found: 432.11210/434.10901; **HPLC** R_T =24.01 min, 99.6% (254 nm), 95.6% (230 nm). Matched previously reported characterisation data however full characterisation data has been reported [2].

Synthesis and Characterisation of 2

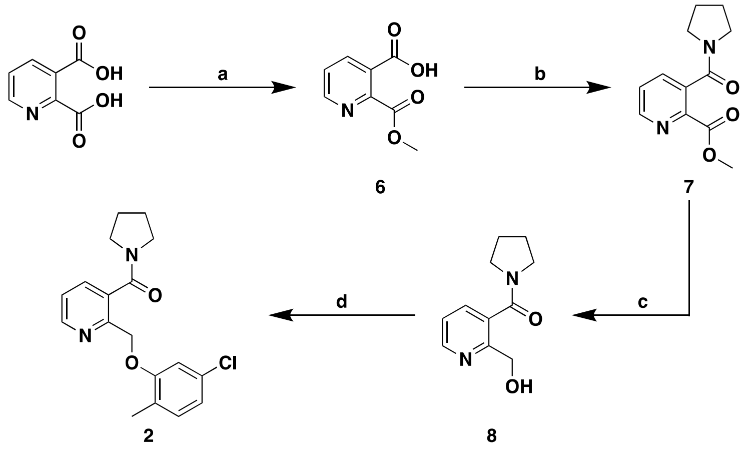

Scheme S2. Synthesis of pyridine core scaffold-hopping derivative **2**. *Reagents and Conditions:* a) *i.* Acetic Anhydride, neat, 120 °C, 5 h *ii.* MeOH, 70 °C, 2 h, 65% (Over 2 Steps) b) Pyrrolidine, EDC·HCl, HOBt, CH_2Cl_2, rt, 30 min, 92% c) $LiBH_4$ (2 M in THF), THF, 0 °C – rt, 1 h, 42% d) *i.* PBr_3, CH_2Cl_2, 0 °C – rt, 12 h *ii.* 5-Chloro-2-methylphenol, NaH, DMF, 0 °C – rt, 30 min, 50% (Over 2 Steps).

2-(Methoxycarbonyl)nicotinic acid (6)

A mixture of quinolinic acid (3.0 g, 18.0 mmol) and acetic anhydride (6.0 mL, 63.5 mmol) was heated at 120 °C for 6 h. The reaction mixture was then concentrated and diluted with CH_2Cl_2 (30 mL). The resulting white precipitate was collected by filtration and washed with CH_2Cl_2 (2 x 10 mL) and dried to yield furo[3,4-*b*]pyridine-5,7-dione as an off-white solid that was used without further purification. A solution of furo[3,4-*b*]pyridine-5,7-dione (2.2 g, 14.8 mmol) in MeOH (30 mL) was heated at reflux for 3 h at which point TLC indicated conversion of the starting material. The reaction mixture was then concentrated under reduced pressure. The crude reaction mixture was recrystalised from EtOAc to yield the title compound as an off-white solid (2.11 g, 65% Over 2 steps). **m.p.** 118 - 122 °C (EtOAc); **^{1}H NMR** (500 MHz, MeOD) δ 8.71 (dd, *J* = 4.9, 1.7 Hz, 1H), 8.36 (dd, *J* = 8.0, 1.6 Hz, 1H), 7.64 (dd, *J* = 8.0, 4.9 Hz, 1H), 3.93 (s, 3H) ppm, COOH proton not observed; **^{13}C NMR** (126 MHz, MeOD) δ 168.72, 167.52, 152.91, 152.71, 139.70, 127.57, 126.42, 53.42 ppm; **LRMS (+ESI)** m/z: 204 ([M+Na]$^+$, 100); **LRMS (-ESI)** m/z: 180 ([M-H]$^-$, 100). Spectral data matched previously reported data [3]. Thermal data matched previously reported data [4].

Methyl 3-(pyrrolidine-1-carbonyl)picolinate (7)

A solution of **6** (502 mg, 2.77 mmol), EDCI (692 mg, 4.46 mmol), HOBt (425 mg, 3.15 mmol) and pyrrolidine (0.25 mL, 3.04 mmol) in CH_2Cl_2 (15 mL) was stirred at r.t. for 30 min, at which point TLC indicated consumption of the starting material. The reaction mixture was then concentrated and purified by flash chromatography (1:19 MeOH/CH_2Cl_2) to yield the title compound as a colourless oil (600 mg, 92%). **R_f** 0.33 (1:19 MeOH/CH_2Cl_2); **^{1}H NMR** (400 MHz, $CDCl_3$) δ 8.74 (dd, *J* = 4.7, 1.7 Hz, 1H), 7.72 (dd, *J* = 7.8, 1.7 Hz, 1H), 7.52 (dd, *J* = 7.8, 4.7 Hz, 1H), 3.97 (s, 3H), 3.68 (t, *J* = 6.9 Hz, 2H), 3.12 (t, *J* = 6.7 Hz, 2H), 2.03 – 1.94 (m, 2H), 1.94 – 1.85 (m, 2H) ppm; **^{13}C NMR** (101 MHz, $CDCl_3$) δ 167.13, 165.19, 149.69, 144.17, 136.19, 135.66, 126.79, 53.28, 48.51, 45.83, 26.02, 24.64 ppm; **LRMS (+ESI)** m/z: 235 ([M+H]$^+$, 12), 257 ([M+Na]$^+$, 63).

(2-(Hydroxymethyl)pyridin-3-yl)(pyrrolidin-1-yl)methanone (8)

A solution **7** (204 mg, mol) in dry THF (8 mL) was cooled to 0 °C and treated dropwise with $LiBH_4$ (2 M in THF, 0.4 mL, 1.02 mmol). The reaction mixture was stirred at 0 °C for 1 h at which point TLC indicated conversion of the starting material. The reaction mixture was quenched by the addition of H_2O (2 mL) and stirred for 15 min. The reaction mixture was then diluted with diethyl ether (10 mL),

dried over $MgSO_4$ and concentrated under reduced pressure. The crude product was purified by flash chromatography (1:19 MeOH/CH_2Cl_2) to yield the title compound as a light-yellow oil (75 mg, 42%). **R*f*** 0.27 (1:19 MeOH/CH_2Cl_2); **^{1}H NMR** (500 MHz, $CDCl_3$) δ 8.60 (dd, *J* = 4.9, 1.7 Hz, 1H), 7.63 (dd, *J* = 7.6, 1.7 Hz, 1H), 7.27 (dd, *J* = 7.7, 4.9 Hz, 1H), 4.75 (s, 2H), 4.16 (br s, 1H), 3.66 (t, *J* = 7.0 Hz, 2H), 3.23 (t, *J* = 6.7 Hz, 2H), 2.03 – 1.94 (m, 2H), 1.94 – 1.86 (m, 2H) ppm; **^{13}C NMR** (126 MHz, $CDCl_3$) δ 167.19, 156.21, 149.13, 134.43, 130.95, 122.14, 63.27, 49.06, 46.00, 26.26, 24.59 ppm; **LRMS (+ESI)** m/z: 207 ($[M+H]^+$, 32), 229 ($[M+Na]^+$, 100).

(2-((5-Chloro-2-methylphenoxy)methyl)pyridin-3-yl)(pyrrolidin-1-yl)methanone (2)

A solution of **8** (70 mg, 339 μmol) in CH_2Cl_2 (5 mL) was put under an atmosphere of N_2 and cooled to 0 °C. The reaction mixture was then treated dropwise with PBr_3 (0.03 mL, 407 μmol). The reaction mixture was warmed to rt and stirred for 30 min, at which point TLC indicated conversion of starting material. The reaction mixture was then added slowly to a solution of sat. aq. $NaHCO_3$ (50 mL). The aqueous phases were then washed with CH_2Cl_2 (3 x 50 mL). Combined organic phases were then washed with brine (1 x 50 mL), dried over $MgSO_4$ and concentrated under reduced pressure to a pink oil. The crude (2-(bromomethyl)pyridin-3-yl)(pyrrolidin-1-yl)methanone was used without further purification. A solution of 5-chloro-2-methylphenol (49 mg, 344 μmol) in DMF (2 mL) was cooled to 0 °C under an atmosphere of N_2 and then treated portionwise with NaH (60% Dispersion in Mineral Oil, 19 mg, 475 μmol). The reaction mixture was stirred for 15 min and then treated dropwise with crude mixture of (2-(bromomethyl)pyridin-3-yl)(pyrrolidin-1-yl)methanone in DMF (2 mL). The reaction mixture was warmed to rt and stirred for 2 h, at which point TLC indicated consumption of starting material. The reaction mixture was diluted in H_2O (50 mL) and extracted with EtOAc (3 x 50 mL). The combined organic phases were then washed with H_2O (1 x 50 mL), 1 M aq. LiCl (1 x 50 mL), brine (1 x 50 mL), dried over $MgSO_4$ and concentrated under reduced pressure. The crude reaction mixture was purified by flash chromatography (1:49 MeOH/CH_2Cl_2) to yield the title compound as a colourless oil that formed a white crystalline solid upon standing (66 mg, 50%). **R*f*** 0.36 (1:49 MeOH/CH_2Cl_2); **^{1}H NMR (**300 MHz, $CDCl_3$) δ 8.66 (dd, *J* = 4.9, 1.7 Hz, 1H), 7.64 (dd, *J* = 7.7, 1.7 Hz, 1H), 7.33 (dd, *J* = 7.7, 4.9 Hz, 1H), 7.01 (dd, *J* = 7.9, 0.9 Hz, 1H), 6.94 (d, *J* = 2.0 Hz, 1H), 6.83 (dd, *J* = 8.0, 2.0 Hz, 1H), 5.29 (s, 2H), 3.61 (t, *J* = 7.0 Hz, 2H), 3.11 (t, *J* = 6.7 Hz, 2H), 2.14 (s, 3H), 1.98 – 1.82 (m, 2H), 1.83 – 1.67 (m, 2H) ppm; **^{13}C NMR** (126 MHz, $CDCl_3$) δ 167.29, 157.39, 153.40, 149.77, 134.79, 133.24, 132.04, 131.36, 125.28, 123.13, 120.95, 112.76, 70.75, 48.93, 45.85, 26.05, 24.57, 15.95 ppm; **LRMS (+ESI)** m/z: 331 ($[M+H]^+$, 32), 353 ($[M+Na]^+$, 100), 683 ($[2M+Na]^+$, 48); **HRMS (+ESI)** Calc. for $C_{18}H_{19}ClN_2O_2$ $[M+H]^+$: 331.1208, found: 331.1202; **HPLC** R_T = 23.57 min, 98.7% (254 nm), 97.71% (230 nm).

References

1. Estévez, V.; Sridharan, V.; Sabaté, S.; Villacampa, M.; Menéndez, J. C., Three-Component Synthesis of Pyrrole-Related Nitrogen Heterocycles by a Hantzsch-Type Process: Comparison between Conventional and High-Speed Vibration Milling Conditions. *Asian J. Org. Chem.* **2016**, *5*, 652-662.
2. Larsen, G. R.; Weigele, M.; Vacca, J. P. Preparation of pyrrolidinecarbonyl-pyrrole- sulfonamides useful in modulation of the inclusion formation treatment of diseases. WO2016090313A1, 2016.
3. He, G.; Chen, G., A Practical Strategy for the Structural Diversification of Aliphatic Scaffolds through the Palladium-Catalyzed Picolinamide-Directed Remote Functionalization of Unactivated C(sp^3) H Bonds. *Angew. Chem. Int. Ed.* **2011**, *50*, 5192-5196.
4. Kenyon, J.; Thaker, K., Ring–chain tautomerism in the acid esters of pyridine-2 : 3-dicarboxylic acid. *J. Chem. Soc.* **1957**, 2531-2536.
